# Efficiency of the healthcare system and the impact of smoking bans: a DEA analysis of the Guangdong-Hong Kong-Macau Greater Bay Area in Asia

**DOI:** 10.3389/fpubh.2025.1576300

**Published:** 2025-04-30

**Authors:** Xiaoyu Wu, Shuyang Wang, Yulong Tang, Jiabi Wang, Jinghua Zhang

**Affiliations:** ^1^School of Business, Macau University of Science and Technology, Macau, China; ^2^School of Accounting, Nanfang College, Guangzhou, Guangdong, China; ^3^Institute of Development Economics, Macau University of Science and Technology, Macau, China

**Keywords:** healthcare system, efficiency, tobacco control, the Greater Bay Area, population aging

## Abstract

**Objective:**

Healthcare system efficiency is a global policy priority in the background of aging populations and in pursuit of universal health coverage (UHC). Some healthcare systems in East Asia have been recognized for being highly efficient, which is attributable to healthy lifestyles, including low smoking rates. Specifically, the Guangdong-Hong Kong-Macao Greater Bay Area (GBA) has offered a unique opportunity to study the link between smoking control and healthcare system efficiency.

**Materials and methods:**

Based on the input and output data from healthcare systems across 11 cities in the GBA between 2010 and 2019, a two-stage output-oriented Data Envelopment Analysis (DEA) was employed to assess healthcare efficiency. Additionally, Tobit regression analysis was conducted to evaluate the determinants of efficiency, including smoking rates, urbanization, population aging, and the proportion of floating populations.

**Results:**

There has been a general trend of improved health production efficiency over the past decade despite fluctuations caused by epidemic shocks. While significant disparities across the region have been identified, Hong Kong and Macao consistently achieved higher efficiency scores compared to other cities in the GBA. The results of the Tobit regression analysis indicate that the coefficients of smoking rates are −1.961 (*p* = 0.000) and −2.134 (*p* = 0.000), respectively, with other socioeconomic confounding factors controlled.

**Conclusion:**

The healthcare systems in the GBA highlight the critical role of smoking control measures in improving healthcare efficiency in terms of population health outcomes. These findings provide evidence-based support not only for the GBA and mainland China but also for other regions aiming to achieve UHC while addressing the health challenges of aging populations.

## 1 Introduction

Healthcare system efficiency refers to the optimal use of resources to provide healthcare services and to achieve desired health outcomes (1–3). In the aftermath of the COVID-19 pandemic, the efficiency of healthcare systems has emerged as a priority for policymakers worldwide to meet the dual challenges of achieving universal health coverage (UHC) and addressing the pressures of aging populations (4).

The efficient performance of healthcare systems in East Asia has garnered considerable attention in recent years. The Bloomberg Health-Efficiency Index (BHE Index) evaluates nations based on life expectancy and health expenditures (5). Hong Kong (China) and Singapore have consistently ranked among the top performers (5), while Macao (China) is estimated to have comparable efficiency (6). In 2020, mainland China was ranked 25th, according to pre-pandemic BHE Index criteria (7). A growing body of research emphasizes the need to examine the determinants of healthcare efficiency in this region, particularly regarding lifestyle factors such as diet and smoking rates (1, 8). Smoking, in particular, remains a globally significant risk factor for non-communicable diseases (NCDs) such as cancer and cardiovascular diseases, which impose substantial economic and social burdens due to direct medical care expenses and indirect costs from productivity losses associated with premature deaths (9–11).

The achievements of Hong Kong's healthcare system can be largely attributed to a unique combination of economic prosperity and low smoking rates (12). Full smoking bans have been rigorously enforced in both Hong Kong and Macao over the past decade (10, 13–15). The implementation of these bans in Macao has led to a significant reduction in smoking rates, dropping from 33.7% in 2011 to 11.2% in 2020 (10). In contrast, although China ratified the WHO Framework Convention on Tobacco Control (FCTC) in 2005, full smoking bans have not yet been adopted, and tobacco control policies remain inconsistent across different cities and regions (16, 17). With the largest tobacco-consuming population in the world (18), the relatively high smoking rates in mainland China undermine its overall health achievements (19, 20).

While existing literature acknowledges that low smoking rates are a key determinant of health outcomes (1, 8, 21, 22) associated with socioeconomic loss (9–11), few studies have empirically analyzed their impacts on healthcare efficiency (23–26). The GBA presents a unique research opportunity due to its shared cultural and socioeconomic characteristics, coupled (27, 28) with divergent policy enforcement—particularly between Hong Kong and Macao (which have comprehensive smoking bans) and mainland Chinese cities (which have inconsistent tobacco control measures). This distinctive contrast enables a novel examination of how varying policy approaches affect healthcare system efficiency within a culturally cohesive region. The majority of residents in the GBA cities share common ethnic backgrounds, dietary habits, and cultural traditions (27, 28). Most GBA cities are characterized by universal healthcare systems (29), with only Macao having a quasi-universal system, where the city government directly provides primary care and the social safety net (10). Over the past decades, the geographical proximity in this region has also fostered active exchanges in cross-border healthcare services and technology (30).

Leveraging Data Envelopment Analysis (DEA), this research provides the first comparative efficiency assessment of healthcare systems across GBA cities, incorporating smoking policy variables as a significant determinant. The findings of this study provide evidence-based recommendations for alleviating the burden of smoking-related diseases and improving healthcare efficiency, with broader implications for public health policies in both developed and developing countries.

## 2 Materials and methods

This study used two stages of efficiency analysis to examine the panel data. First, Data Envelopment Analysis (DEA) was employed to evaluate the healthcare system performance of 11 cities. Second, a Tobit regression model was applied to analyze related factors that affect healthcare system efficiency.

### 2.1 Statistical method

#### 2.1.1 Data envelop analysis (DEA) method

DEA is widely utilized in the healthcare sector for its ability to establish a deterministic relationship between resource inputs and health outputs. This linear programming technique considers multiple inputs and outputs simultaneously (31–34). It calculates a relative efficiency score for decision-making units (DMUs) by optimizing the allocation of inputs and outputs. In this context, DMUs refer to cities evaluated on a scale from 0 (least efficient) to 1 (most efficient) (35–37). Efficient DMUs form a production frontier that serves as a benchmark for all inefficient DMUs. Efficiency is the weighted sum of outputs compared to the weighted sum of inputs, indicating how effectively different areas convert inputs into outputs (25, 38–41).

The mathematical representation of the DEA approach is as follows (42):


(1) $$\begin{array}{l} Max.E_{q}\frac{\sum_{i=1}^{r}u_{i}y_{iq}+\;u_{0}}{\sum_{j=1}^{m}v_{j}x_{jq}}\\ s.t.\;\frac{\sum_{i=1}^{r}u_{i}y_{iq}+\;u_{0}}{\begin{array}{c} \sum_{j=1}^{m}v_{j}x_{jq}\leq 1;\left(q=1,2,\ldots ,n\right)\\ u_{i,\;}v_{j}\geq \varepsilon >0;\;u_{0\;}\;\varepsilon \;R \end{array}} \end{array}$$

In Equation 1, *E*_*q*_ is the efficiency score of DMU*q*; the greater the value, the more effective DMU*q*; *y*_*iq*_ denotes the value of output *i* of DMU*q*, and *x*_*jq*_ represents the value of input *j* of DMU*q*. In addition, *r* denotes the number of outputs, *m* denotes the number of inputs, *u*_*i*_, and *v*_*j*_ are the weights assigned by the DEA to output *i* and input *j*, respectively, to determine the level of efficiency, and *n* stands for the number of DMUs included in the sample.

Based on the assumption of constant returns to scale (CRS) in production, a specific form of the DEA model developed by Charnes, Cooper, and Rhodes (CCR) offers insights into the relationship between input and output (43, 44). This model suggests that any change in the input will result in a proportional change in the output, which is also called input-oriented (45, 46). It aims to minimize input resources while maintaining a constant level of output (47–49). On the other hand, a different model developed by Banker, Charnes, and Cooper (BCC) considers variable returns to scale (VRS), meaning that increasing the input can lead to either an increase or a decrease in the output (50, 51). This approach is particularly valuable for this study as it aims to evaluate the efficiency of different organizational units (such as healthcare systems in various cities) that utilize diverse resources to produce multiple outputs (42, 52–54). This perspective provides a more accurate and realistic representation of changes in real-world scenarios, such as evaluating the healthcare system in a specific area (55). This is also called the output-oriented approach, which focuses on maximizing output while keeping input resources constant (48).

This study chooses the BCC model because of the inherent differences in health output within the healthcare system. Moreover, considering the dynamic nature of health levels across different locations, it is unlikely for them to remain constant (32). Furthermore, the objective of the health production system is to attain optimal output levels, accounting for the constraints posed by the available financial budget (48).

Additionally, this paper also applied the DEA-Malmquist index approach. DEA models have inherent limitations, such as they can only analyze time series and cross-sectional data, and they are unable to adequately capture the dynamic variations in the efficiency of DMUs (47, 56). The utilization of the Malmquist index as a frontier analysis technique can improve the above limitations because it can facilitate the systematic evaluation of DMUs on an annual basis, providing a comprehensive assessment of changes in their overall factor productivity (49). When combining the DEA and Malmquist models together, a comprehensive efficiency analysis can be conducted, offering both a static observation of efficiency scores for a specific year and a dynamic observation of consecutive year efficiency change scores. This combined approach allows for a more comprehensive understanding of the efficiency trends over time. The assessment of total factor productivity change (TFP) involves the decomposition into two key components: favorable technology advancement (TECHCH) and technical efficiency change (EFFCH). Moreover, the EFFCH component can be further subdivided into two distinct factors: pure efficiency (PECH) and scale efficiency (SECH) (57, 58).

The following equation shows the DEA-Malmquist index approach (59):


(2) $$\begin{array}{l} M_{i}^{t+1}\left(y^{t+1},x^{t+1},y^{t},x^{t}\right)=\\ \;{\left[\frac{d_{i}^{t}\left(y^{t+1},x^{t+1}\right)}{d_{i}^{t}\left(y^{t},x^{t}\right)}\times \frac{d_{i}^{t+1}\left(y^{t+1},x^{t+1}\right)}{d_{i}^{t+1}\left(y^{t},x^{t}\right)}\right]}^{\frac{1}{2}} \end{array}$$

In the above formula, *x* and *y* represent the input and output vectors. $d_{i}^{t}\left(y^{t},x^{t}\right)$ and $d_{i}^{t}\left(y^{t+1},x^{t+1}\right)$ are the distance functions in period *t* and period *t*+*1*. If the result TFP index exceeds 1, it indicates an increase in TFP from period t to t+1. Conversely, if the index is < 1, it signifies a decrease in TFP over the same duration (60). You may insert up to 5 heading levels into your manuscript, as can be seen in the “Styles” tab of this template. These formatting styles are meant as a guide. As long as the heading levels are clear, the Frontiers style will be applied during typesetting.

#### 2.1.2 Tobit regression

One limitation of the DEA approach is the presence of serial correlation, which is caused by the correlation between inputs and outputs in the efficiency scores (31). To account for this limitation, a two-stage analysis is recommended, wherein the explanatory variables influencing healthcare system efficiency obtained from DEA in the first stage are further examined in the second stage using econometric models (31). This study is based on the efficiency score results from the BCC model as dependent variables. Nevertheless, it is crucial to acknowledge that the efficiency scores derived from the DEA model possess a censored structure, rendering the use of parametric estimations such as ordinary least squares (OLS) regression in the second stage biased and inconsistent. To overcome this issue, a more comprehensive approach is necessary, employing a Tobit regression model, which best fits the situation when the variables assume the limiting values and have a lower or upper limit in the second stage instead of the traditional panel analysis (31, 61–65).

A Tobit model was employed to examine the efficiency of the GBA in the healthcare system. A positive estimated regression coefficient shows a favorable impact of the respective factor on efficiency, while a negative coefficient suggests a detrimental effect on efficiency. The Tobit regression model was formulated as follows:


(3) $$y_{it}=\{\begin{array}{c} \beta^{T}x_{it}+\varepsilon_{it}\;>0\\ \beta^{T}x_{it}+\varepsilon_{it}\;\leq 0 \end{array}$$

In the specified Tobit regression model, the dependent variable *y*_*it*_denotes the efficiency score from DEA-BCC. When the efficiency score is >0, it takes on its actual observed value, but when it is equal to or < 0, it is constrained and set to 0. The explanatory variables are representing as *x*_*it*_. The vector of parameters to be estimated is indicated as β^*T*^. Additionally, ε_*it*_ denotes the stochastic error term.

In addressing potential autocorrelation, which can occur due to the correlation between inputs and outputs in the efficiency scores, we utilized random effects estimation in our panel Tobit analysis, following the recommendations of Samut and Cafri (31). They point out that the non-linear nature of the Tobit model can lead to incidental parameter problems when applying fixed effects, resulting in biased estimates (66). Greene also highlights that fixed-effect Tobit models may face challenges with the distribution of disturbance variance estimators, further complicating the incidental parameters issue (67).

To address heteroscedasticity, we implemented several strategies. First, we used a heteroscedasticity-robust Tobit model, which employs maximum likelihood estimators that explicitly account for heteroscedasticity. Additionally, we applied bootstrapping to the DEA efficiency scores and Tobit regression to address the inherent variability in DEA. Finally, we log-transformed the skewed variable of GDP per capita to reduce issues related to non-constant variance. Together, these methodologies enhance the robustness of our analysis.

### 2.2 Data source and variables

#### 2.2.1 Data source

The dataset analyzed in this study was mainly obtained from the Health Statistical Yearbook (2010–2019) and the Statistics Yearbook (2010–2019) for the 11 cities in the GBA, China. The smoking rates in each city were collected from the respective Health Bureaus. This study focused on the years before 2020 to avoid the potential confounding effects of the Covid-19 pandemic.

#### 2.2.2 Output variables for the DEA model

##### 2.2.2.1 Population mortality rate

Health outcome measurements—such as population mortality rates and life expectancy at birth—are widely used indicators of health system performance in Data Envelopment Analysis (DEA) models (1, 4, 8, 68).

The output variable in this study is the population mortality rate, also known as the crude death rate (23, 69). The population mortality rate measures the number of deaths recorded in a specific calendar year per 1,000 individuals within the mid-year population of that same year (4, 70). Since the correlation between population mortality rate and healthcare efficiency is negative, the mortality rate decreases when a healthcare system is closer to its efficient state. Therefore, this study adopts the inverse of the population mortality rate in the model (71). The mortality rates in Hong Kong and Macao were age-adjusted based on the population structure of Guangdong Province (72).

This study utilizes mortality rates as the output variable instead of life expectancy, primarily due to data limitations (73). Life expectancy in China is updated only once every 5 years, whereas mortality data is available annually. Moreover, mortality rates provide a timely measure of ultimate health outcomes, despite potential yearly fluctuations.

#### 2.2.3 Input variables of the DEA model

Inputs were defined as resources required to facilitate the production function of the health system. The majority of the previous studies categorize inputs into three categories: health system building blocks, social determinants of health, and health risk factors (1, 25). Health system building blocks were the ones being considered the most. It includes finances such as annual GDP, total health expenditure as a percentage of GDP, and so on (1, 68, 74), human resources for health, which comprise the number of health workers per 1,000 population, the number of specialists or resident medical specialists per 100,000, and so on (1, 32, 57), physical medical infrastructures such as total number of hospital beds (1, 62, 74).

Three inputs have been chosen in this study:

*Hospital beds* are considered to be physical medical infrastructure. The number of hospital beds per 1,000 population is the available hospital beds for every 1,000 people in a particular population (75, 76).
*The number of physicians per 1,000 population* reflects the input of healthcare human resources. This includes both generalist and specialty practitioners (57, 77).
*Total health expenditure* represents a percentage of GDP, reflecting the portion of Gross Domestic Product (GDP) allocated to healthcare (74). It measures the financial resources directed toward enhancing and supporting the provision of healthcare services (57).

#### 2.2.4 Explanatory variables for the Tobit model

Human health is mainly influenced by economic, social, environmental, and cultural factors (78). Income distribution, income level, or country/sub-national income levels, such as GDP per capita, are examples of economic categories (1, 79). Social, environmental, and cultural factors include population density, rural-urban population distribution, and age structure (62), which reflect population characteristics and lifestyle risk factors, such as tobacco use (1, 62, 80).

This study chooses the following four factors as explanatory variables to explore how these variables affect the healthcare efficiency of 11 cities.

##### 2.2.4.1 GDP per capita

GDP per capita is included to control for economic development in a region (21, 79, 81), which is linked to government investment in healthcare and improvements in the efficiency of the healthcare system (76). GDP per capita was log-transformed to address its right-skewed distribution and to enhance the assumptions of linearity.

##### 2.2.4.2 Urbanization rate

The urbanization rate is calculated by dividing the total urban population by the total population living in the area (80, 82).

##### 2.2.4.3 Smoking rate

The smoking rate was calculated based on the ratio of current adult smokers (aged 18 years or older) to the population (10). Due to the inconsistent disclosure of smoking rates across nine cities in Guangdong Province, China, the smoking rates in this study were estimated using an arithmetic sequence over 10 years. Since Hong Kong and Macau reported smoking rates biennially between 2010 and 2019, any missing data for these two cities were imputed using the mean value of the 2 nearest years.

##### 2.2.4.4 Population aging rate

Aging is a key determinant of resource allocation in the healthcare system (83, 84), as older adult individuals typically require greater medical care (85). In this study, the population aging rate is defined as the proportion of individuals aged 60 and above within the total population of a city (86). This threshold is used because 60 is the official retirement age for men in China, and the nine cities in Guangdong Province report aging rates based on this age group. The most recent population aging rates for these nine cities were disclosed in 2015. However, this data remains valid for analysis, as the aging rate typically exhibits minimal variation over a 5-year period.

##### 2.2.4.5 Proportion of the floating population

In China, the term “floating population” refers to individuals whose current places of residence differ from their officially registered ones (87, 88), effectively representing domestic migrants. When a city experiences a higher proportion of either inflow or outflow of migrants, adjustments to the efficiency of the healthcare system become necessary. The floating population is calculated as the ratio of the floating population to the total population of a city (88).

Definitions for all variables are provided in Table 1.

**Table 1:** Definition of variables.

**Category**	**Variable**	**Definition**	**References**
Output variable	Population mortality rate	The number of deaths recorded in a specific calendar year per 1,000 individuals within the mid-year population of that same year, the inverse of the population mortality rate	(70, 71)
Input variables	Hospital beds per 1,000 population	The number of hospital beds that are available per 1,000 people in a certain population	(75)
	Physicians per 1,000 population	The number of physicians per 1,000 people	(77)
	Total health expenditure	Percentage of GDP reflects the allocation of a portion of Gross Domestic Product (GDP) toward healthcare	(57)
Explanatory variables (Tobit)	GDP Per Capita	Gross domestic product per capita (RMB¥)	(76)
	Urbanization rate	Total urban population over the total population living in the area	(80, 82)
	Smoking rate	The ratio of current adult smokers (aged 18 years or older) to the population	(10)
	Population aging rate	The proportion of individuals aged 60 and above within the total population of a city	(86)
	Proportion of the floating population	The ratio of the floating population to the total population of a city	(87, 88)

## 3 Results

### 3.1 Descriptive statistics

The descriptive statistics in Table 2 highlight key differences among cities within the GBA. The average population mortality rate is significantly lower in Hong Kong and Macao (1.345) compared to the nine cities in Guangdong Province (4.600), indicating better health outcomes in the former. Meanwhile, Hong Kong and Macao have higher health expenditures as a percentage of GDP (2.1% vs. 0.9%), suggesting that greater financial resources are allocated to health. However, the nine cities in Guangdong have slightly more hospital beds and physician availability, indicating a stronger healthcare infrastructure relative to population size.

**Table 2:** Descriptive statistics of input, output, and explanatory variables.

	**Total**		**Hong Kong and Macao**		**Nine cities in Guangdong**	
**Variables**	**Mean**	**SD**	**Mean**	**SD**	**Mean**	**SD**
**Output variable**						
Population mortality rate (per 1,000 population)	3.76	2.66	1.345	0.232	4.600	1.984
**Input variables**						
Total health expenditure/GDP	0.011	0.008	0.021	0.008	0.009	0.005
Hospital beds per 1,000 population	3.981	1.122	3.752	1.484	4.032	1.028
Physicians per 1,000 population	2.312	0.638	2.243	0.370	2.328	0.684
**Explanatory variables**						
GDP per capita (10 k RMB)	14.555	13.063	38.333	13.155	9.272	4.093
Ln (GDP per capita) (10 k RMB)	11.603	0.718	12.799	0.350	11.337	0.460
Population aging rate	0.104	0.034	0.131	0.028	0.099	0.033
Floating population rate	0.431	0.316	0.530	0.482	0.409	0.264
Smoking rate	0.211	0.052	0.125	0.026	0.239	0.046
Urbanization rate	0.839	0.170	1.00	0.00	0.803	0.1689
Observations	110		20		90	

Among other explanatory variables, the Hong Kong and Macao regions have a higher aging population (13.1% vs. 9.9%), urbanization rate (100% vs. 80.3%), and floating population rate (53.0% vs. 40.9%). However, on average, the two cities report a lower smoking rate (12.5% vs. 23.9%).

### 3.2 Results from the DEA and DEA-Malmquist Index approach

**Figure 1:**
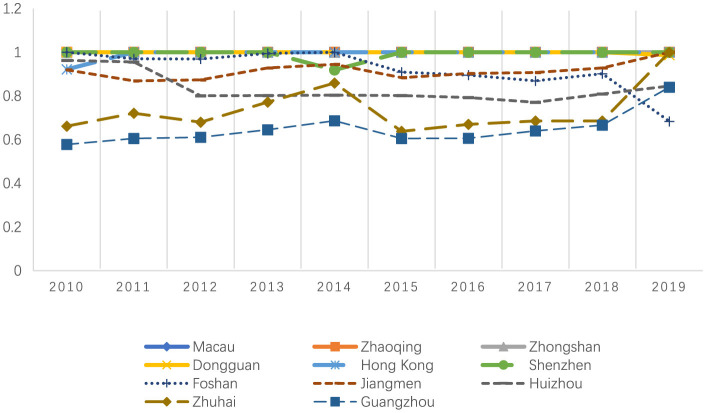
DEA-BCC by year.

Table 3, Figure 1 present a comprehensive annual analysis of individual cities using the DEA-BCC model. The mean efficiency scores across all DMUs show a general upward trend, increasing from 0.913 in 2010 to 0.942 in 2019, with the lowest efficiency score of 0.894 recorded in 2015.

**Table 3:** DEA-BCC by year, 2010–2019.

**DMUs**	**2010**	**2011**	**2012**	**2013**	**2014**	**2015**	**2016**	**2017**	**2018**	**2019**	**Mean**
Macau	1.000	1.000	1.000	1.000	1.000	1.000	1.000	1.000	1.000	1.000	1.000
Zhaoqing	1.000	1.000	1.000	1.000	1.000	1.000	1.000	1.000	1.000	1.000	1.000
Zhongshan	1.000	1.000	1.000	1.000	1.000	1.000	1.000	1.000	1.000	1.000	1.000
Dongguan	1.000	1.000	1.000	1.000	1.000	1.000	1.000	1.000	1.000	0.987	0.999
Hong Kong	0.922	1.000	1.000	1.000	1.000	1.000	1.000	1.000	1.000	1.000	0.992
Shenzhen	1.000	1.000	1.000	1.000	0.919	1.000	1.000	1.000	1.000	1.000	0.992
Foshan	1.000	0.970	0.969	0.995	1.000	0.909	0.896	0.869	0.902	0.684	0.919
Jiangmen	0.919	0.869	0.874	0.928	0.945	0.884	0.903	0.907	0.928	1.000	0.916
Huizhou	0.963	0.956	0.801	0.802	0.804	0.802	0.793	0.771	0.809	0.846	0.835
Zhuhai	0.662	0.721	0.680	0.772	0.859	0.638	0.670	0.685	0.685	1.000	0.737
Guangzhou	0.578	0.605	0.611	0.645	0.686	0.605	0.606	0.640	0.666	0.840	0.648
Mean	0.913	0.920	0.903	0.922	0.929	0.894	0.897	0.898	0.908	0.942	0.913

High-performing DMUs, including Hong Kong, Macau, Dongguan, Shenzhen, Zhaoqing, and Zhongshan, consistently achieved full efficiency (1.000) across most or all years. Moderate-performing DMUs, including Foshan, Jiangmen, and Huizhou, exhibited inefficiency and significant fluctuations during the studied period. It is noteworthy that Guangzhou and Zhuhai had low performance despite being developed central cities in the GBA. Guangzhou's efficiency scores were relatively low compared to other DMUs, starting at 0.578 in 2010 and peaking at 0.840 in 2019.

**Figure 2:**
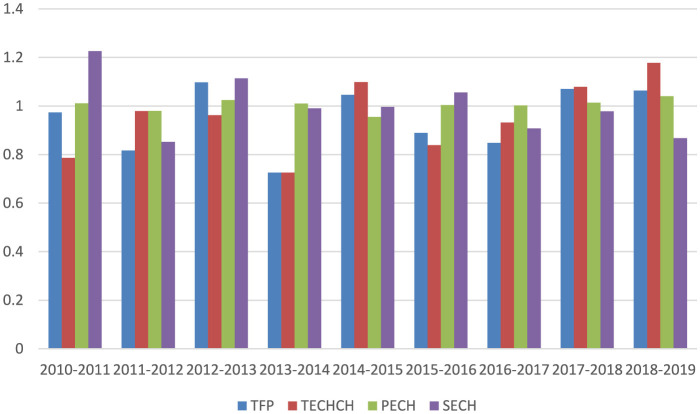
The Malmquist index and its decomposition for the GBA.

The first column of Table 4 shows that, on average, the TFP score for the GBA from 2010 to 2019 was 0.948, suggesting an overall decline in healthcare productivity. During this period, TFP scores fluctuated significantly—from a low of 0.726 during 2013–2014 to a peak of 1.098 in 2012–2013 (refer to Figure 2). Similarly, the average TECHCH score was 0.953, indicating a modest decline in technological progress throughout the decade, with its pattern closely mirroring that of TFP. Meanwhile, the average PECH score was 1.005, as values remained near 1 in most years, signaling only minimal gains in pure efficiency. Finally, the SECH score had a mean of 0.999, indicating almost no change in scale efficiency over the period.

**Table 4:** The Malmquist index and its decomposition for the GBA, 2010–2019.

**Period**	**TFP**	**TECHCH**	**PECH**	**SECH**
2010–2011	0.974	0.786	1.011	1.226
2011–2012	0.817	0.979	0.980	0.852
2012–2013	1.098	0.962	1.025	1.114
2013–2014	0.726	0.726	1.010	0.991
2014–2015	1.046	1.099	0.955	0.996
2015–2016	0.889	0.839	1.004	1.056
2016–2017	0.848	0.932	1.002	0.908
2017–2018	1.070	1.079	1.014	0.978
2018–2019	1.064	1.178	1.041	0.868
Mean	0.948	0.953	1.005	0.999

**Figure 3:**
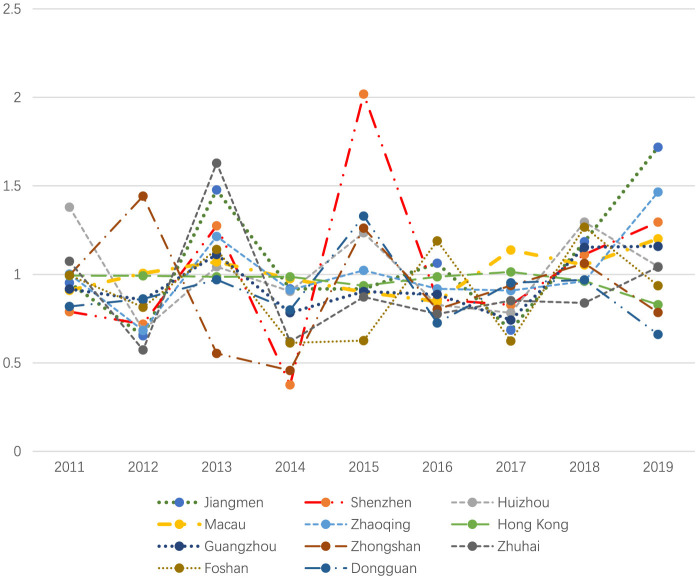
The TFP under the Malmquist index for the 11 cities in GBA.

Table 5 summarizes Total Factor Productivity (TFP) using the Malmquist index. As illustrated in Figure 3, some PMUs demonstrated strong and consistent productivity growth, while others experienced stagnation or high volatility. High performers include cities such as Jiangmen (mean TFP: 1.063), Shenzhen (1.030), Huizhou (1.022), Macau (1.011), and Zhaoqing (1.010), all demonstrating productivity growth. In contrast, Hong Kong (0.965), Guangzhou (0.946), and Foshan (0.912) faced some volatility, but overall maintained stable or improving trends. Finally, Dongguan (0.898), Zhongshan (0.922), and Zhuhai (0.920) experienced fluctuations and lagged behind.

**Table 5:** The TFP under the Malmquist index for the 11 cities in GBA, 2010–2019.

**DMUs**	**2011**	**2012**	**2013**	**2014**	**2015**	**2016**	**2017**	**2018**	**2019**	**Mean**
Jiangmen	0.953	0.653	1.477	0.918	0.917	1.063	0.685	1.185	1.719	1.063
Shenzhen	0.789	0.719	1.275	0.376	2.019	0.858	0.827	1.113	1.295	1.030
Huizhou	1.380	0.685	1.043	0.904	1.235	0.828	0.783	1.295	1.045	1.022
Macau	0.910	1.006	1.071	0.975	0.900	0.847	1.138	1.053	1.201	1.011
Zhaoqing	1.002	0.683	1.214	0.912	1.023	0.918	0.909	0.961	1.465	1.010
Hong Kong	0.993	0.992	0.987	0.987	0.936	0.986	1.014	0.962	0.829	0.965
Guangzhou	0.917	0.856	1.112	0.784	0.903	0.887	0.742	1.153	1.159	0.946
Zhongshan	0.995	1.442	0.554	0.457	1.262	0.802	0.937	1.062	0.785	0.922
Zhuhai	1.074	0.573	1.629	0.619	0.873	0.776	0.852	0.839	1.041	0.920
Foshan	0.995	0.814	1.141	0.613	0.626	1.189	0.624	1.267	0.936	0.912
Dongguan	0.819	0.861	0.970	0.799	1.330	0.726	0.953	0.969	0.661	0.898

**Figure 4:**
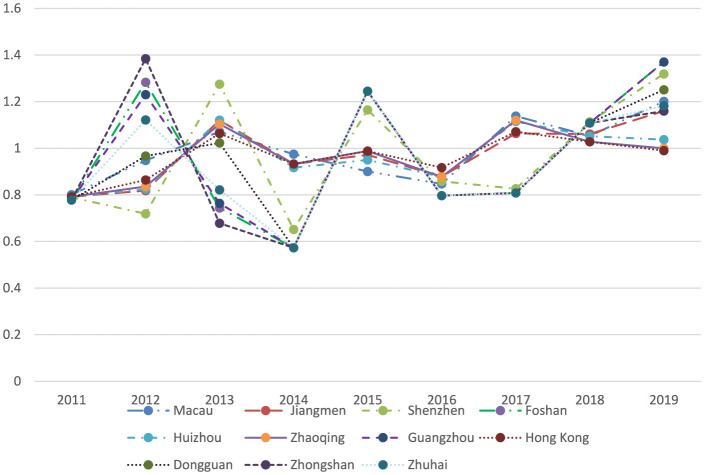
The TECHCH under the Malmquist index for the 11 cities in GBA.

As depicted in Table 6, Figure 4, the scores of TECHCH under the Malmquist index reveal that Macau achieved the highest mean value of 0.993, indicating strong performance during the studied period. In contrast, Zhuhai recorded the lowest mean of 0.937, reflecting less favorable outcomes. Overall, the majority of cities displayed fluctuations in their performance, but Macau and Jiangmen stood out for demonstrating consistent growth throughout the years.

**Table 6:** The TECHCH under the Malmquist index for the 11 cities in GBA, 2010–2019.

**DMUs**	**2011**	**2012**	**2013**	**2014**	**2015**	**2016**	**2017**	**2018**	**2019**	**Mean**
Macau	0.801	0.948	1.071	0.975	0.900	0.847	1.138	1.053	1.201	0.993
Jiangmen	0.792	0.818	1.120	0.933	0.972	0.879	1.064	1.061	1.160	0.978
Shenzhen	0.789	0.719	1.275	0.652	1.165	0.858	0.827	1.113	1.319	0.968
Foshan	0.778	1.283	0.744	0.573	1.245	0.797	0.808	1.108	1.370	0.967
Huizhou	0.792	0.824	1.121	0.917	0.950	0.877	1.115	1.052	1.037	0.965
Guangzhou	0.778	1.230	0.764	0.573	1.245	0.797	0.808	1.108	1.370	0.964
Zhaoqing	0.792	0.836	1.104	0.933	0.988	0.877	1.120	1.028	1.000	0.964
Hong Kong	0.792	0.864	1.064	0.933	0.988	0.917	1.071	1.028	0.990	0.961
Dongguan	0.778	0.967	1.023	0.573	1.245	0.797	0.808	1.108	1.251	0.950
Zhongshan	0.778	1.384	0.678	0.573	1.245	0.797	0.808	1.108	1.160	0.948
Zhuhai	0.778	1.122	0.822	0.573	1.245	0.797	0.808	1.108	1.182	0.937

### 3.3 Result from Tobit regression

Based on the efficiency scores obtained from the DEA-BCC model, a Tobit regression analysis was conducted. As reported in Table 7, the coefficients for smoking rates are highly significant and negative in both models: −1.961 (*p* < 0.01) in Model (1) and −2.134 (*p* < 0.01) in Model (2). This indicates that higher smoking rates are strongly associated with lower health production efficiency, as measured by the Efficiency Score from the DEA-BCC model. The magnitude of the coefficients suggests that smoking has a substantial detrimental effect on health production efficiency.

**Table 7:** Result from Tobit regression.

**Tobit regression^a^**	**Model (1)**		**Model (2)**	
**Variables**	**Regression coefficient** ^b^	***P*** **-value**	**Regression coefficient**	***P*** **-value**
Ln (GDP per capita)	−0.053	0.255	0.033	0.615
Smoking rate	−1.961^***c^	0.000	−2.134^***^	0.000
Population aging rate	−2.404^**^	0.019	−1.585^***^	0.047
Floating population rate	−0.301^**^	0.035		
Urbanization rate			−0.005^*^	0.059
Constant	2.423^***^	0.000	1.715^***^	0.013
Observations	110	110

^a^Dep. var. = Efficiency Score from DEA-BCC model.

^b^Bootstrapped 1,000 times. Robust standard errors are shown in parentheses.

^c^^***^p < 0.01, ^**^p < 0.05, ^*^p < 0.1.

The Population Aging Rate has negative coefficients (−2.404 and −1.585, *p* < 0.05 and *p* < 0.01, respectively), indicating that an aging population reduces the efficiency of health production. The floating population rate has a negative coefficient (−0.301, *p* < 0.05), suggesting its association with lower efficiency. Conversely, the coefficients of Ln (GDP Per Capita) are insignificant in both models.

The urbanization rate was included as an alternative variable for a robustness check because it correlates with the floating population rate (e.g., rural-to-urban migrants). The coefficient for the urbanization rate is negative and statistically significant at the 10% level (−0.005, *p* < 0.1).

## 4 Discussion

The analysis of the healthcare system's efficiency performance based on the DEA approach indicates a general trend of improved health production efficiency over the past decade, despite fluctuations caused by external shocks. Notably, the most significant interruption in this upward trajectory occurred during the Dengue Fever outbreak in 2013 and 2014 (89), which was followed by increased public health expenditure (90) and led to a marked decline in efficiency in 2015 (see Table 2). Similarly, the avian influenza outbreaks in 2018 and 2019 (91–93) further contributed to fluctuations in Total Factor Productivity (TFP), as illustrated in Tables 3, 4.

Moreover, the favorable results in the Pure Efficiency Change (PECH) component for 10 out of the 11 cities (Table 3) suggest significant improvements in managerial skills and resource allocation, which can be attributed to investments in human capital and enhanced management practices throughout the GBA.

The findings of this study reveal significant disparities in healthcare efficiency among member cities within the GBA. Economic differences such as income inequality, regional wealth gaps, and the city's population structure, comprising urban and migrant populations, along with the distribution of healthcare infrastructure and resources, including the concentration of top hospitals, as well as government policies and funding, are the main causes of these disparities in healthcare efficiency (94, 95). Compared to Zhongshan, which effectively utilizes its community health centers and allocates its primary healthcare resources due to a relatively balanced population distribution (96), Huizhou's high-quality medical resources are disproportionately concentrated in core urban areas. As a result, municipal hospitals are tasked with managing a high volume of common diseases that could otherwise be addressed by primary care facilities (97). In Zhuhai's western region, with its agrarian economy, traditional manufacturing base, and sparse population, policymakers have historically focused development efforts on the eastern part (98). However, even after significant recent investments in the West, cross-regional healthcare-seeking behavior among residents persists, exacerbating inefficiencies in local medical resource allocation (99). High-income, well-governed regions such as Hong Kong and Macau consistently outperform others, such as Guangzhou, a major central city in South China.

When examining the efficiency of the Chinese healthcare system or institutions, most current literature measures output in terms of healthcare service provision and reports that these institutions generally meet efficiency benchmarks (100, 101). However, this study highlights that, despite its advanced healthcare technology and skilled workforce, Guangzhou, as the central city in South China, did not perform optimally based on population mortality metrics. Two potential explanations for this underperformance are proposed. First, the efficiency metrics may be underestimated due to the presence of nonlocal medical tourists and domestic migrants, whose health outcomes are not captured in local efficiency evaluations. Many patients prefer to seek care in accredited or high-ranking hospitals in major cities (102), which complicates the assessment of local healthcare efficiency. The lack of official data on migrant healthcare utilization presents a challenge that warrants further investigation. Second, inefficiencies in Guangzhou may stem from overtreatment or the wasteful use of healthcare resources (103, 104). The ongoing healthcare reforms in China, aimed at cost containment and combating medical corruption, seek to enhance the value-based efficiency of the healthcare system for long-term sustainability (105, 106).

The Tobit regression analysis identifies smoking rates as a significant determinant of healthcare system efficiency, with higher smoking rates correlating with a decrease in efficiency scores of approximately two points. In other words, cities with lower smoking rates, such as Hong Kong and Macao, which have implemented comprehensive smoking bans, consistently demonstrate higher levels of efficiency scores. This finding aligns with existing literature on the determinants of longevity and lower mortality rates in Hong Kong and Macao (12, 107–109).

The estimated coefficient for the population aging rate is −2.404 (*p* = 0.019), indicating that each incremental year in the mean age of the older adult population is associated with a 2.4-point reduction in systemic efficiency scores. This finding aligns with the current literature (83, 84). Additionally, the findings of this study reveal that the relationship between urbanization and healthcare system efficiency in newly industrialized regions is complex and multifaceted (110). As highlighted in the literature, during the process of urbanization in emerging economies such as China, improved access to healthcare services and higher income levels in urban areas may drive demand for healthcare, while more advanced medical infrastructure and higher costs of living there tend to significantly increase healthcare expenditure (102, 111). In addition, the newly urbanized populations may face a higher prevalence of non-communicable diseases, which are more costly to treat (111).

This study has some limitations. Due to data availability, the DEA model's output indicators for healthcare efficiency are limited to the population mortality rate, excluding other important metrics. Additionally, this study focuses on the Greater Bay Area (GBA), the most developed region in China. Future empirical research will expand the analysis to include comparable cities nationwide, thereby enhancing the estimation of policy effects. In addition, potential confounding factors, such as advancements in medical technology and improvements in the treatment of underlying diseases, may influence the outcomes. Further research is required to provide more comprehensive insights into the long-term effectiveness of these measures.

## 5 Conclusion

This study highlights the significant disparities in efficiency among healthcare systems in the GBA region, revealing that Hong Kong and Macao demonstrate consistent efficiency performance due to their lower smoking rates while controlling for other confounding socioeconomic factors.

The findings of this study have enriched the empirical evidence for policymakers seeking to improve healthcare system efficiencies, not only within the GBA and mainland China but also in other emerging economies worldwide. These policy implications are particularly relevant for regions aiming to achieve universal health coverage and address the healthcare challenges posed by aging populations and lifestyle-related health risks.

## Data Availability

The datasets presented in this study can be found in online repositories. The names of the repository/repositories and accession number(s) can be found below: https://www.gdhealth.net.cn/ebook/2019nianjian/mobile/index.html#p=1.
