# Supplementary material for: Efficiency of the healthcare system and the impact of smoking bans: a DEA analysis of the Guangdong-Hong Kong-Macau Greater Bay Area in Asia
Source: Front Public Health. 2025 Apr 30;13:1576300. doi: 10.3389/fpubh.2025.1576300 (PMC12075119; doi:10.3389/fpubh.2025.1576300)
Supplement: Supplementary file 1 [file Table_1.docx]

Supplementary Material

# Supplementary Figures and Tables

Supplementary Table 1: The PECH under Malmquist index for the 11 cities in GBA, 2010-2019

| DMUs | 2011 | 2012 | 2013 | 2014 | 2015 | 2016 | 2017 | 2018 | 2019 | Mean |
| --- | --- | --- | --- | --- | --- | --- | --- | --- | --- | --- |
| Zhuhai | 1.090 | 0.943 | 1.135 | 1.114 | 0.742 | 1.051 | 1.023 | 1.000 | 1.460 | 1.062 |
| Guangzhou | 1.048 | 1.010 | 1.055 | 1.064 | 0.881 | 1.002 | 1.056 | 1.042 | 1.263 | 1.047 |
| Jiangmen | 0.946 | 1.005 | 1.062 | 1.019 | 0.935 | 1.022 | 1.005 | 1.022 | 1.078 | 1.010 |
| Hong Kong | 1.085 | 1.000 | 1.000 | 0.919 | 1.089 | 1.000 | 1.000 | 1.000 | 1.000 | 1.009 |
| Shenzhen | 1.000 | 1.000 | 1.000 | 0.919 | 1.089 | 1.000 | 1.000 | 1.000 | 1.000 | 1.001 |
| Macau | 1.000 | 1.000 | 1.000 | 1.000 | 1.000 | 1.000 | 1.000 | 1.000 | 1.000 | 1.000 |
| Zhaoqing | 1.000 | 1.000 | 1.000 | 1.000 | 1.000 | 1.000 | 1.000 | 1.000 | 1.000 | 1.000 |
| Zhongshan | 1.000 | 1.000 | 1.000 | 1.000 | 1.000 | 1.000 | 1.000 | 1.000 | 1.000 | 1.000 |
| Dongguan | 1.000 | 1.000 | 1.000 | 1.000 | 1.000 | 1.000 | 1.000 | 1.000 | 0.987 | 0.999 |
| Huizhou | 0.993 | 0.838 | 1.001 | 1.002 | 0.998 | 0.989 | 0.973 | 1.049 | 1.045 | 0.988 |
| Foshan | 0.970 | 0.999 | 1.027 | 1.005 | 0.909 | 0.986 | 0.970 | 1.037 | 0.759 | 0.962 |

| DMUs | 2011 | 2012 | 2013 | 2014 | 2015 | 2016 | 2017 | 2018 | 2019 | Mean |
| --- | --- | --- | --- | --- | --- | --- | --- | --- | --- | --- |
| Huizhou | 1.754 | 0.992 | 0.929 | 0.983 | 1.304 | 0.954 | 0.722 | 1.173 | 0.965 | 1.086 |
| Jiangmen | 1.271 | 0.793 | 1.243 | 0.966 | 1.009 | 1.184 | 0.641 | 1.093 | 1.375 | 1.064 |
| Zhaoqing | 1.265 | 0.817 | 1.100 | 0.977 | 1.035 | 1.047 | 0.811 | 0.935 | 1.465 | 1.050 |
| Foshan | 1.318 | 0.635 | 1.492 | 1.063 | 0.554 | 1.513 | 0.796 | 1.102 | 0.901 | 1.042 |
| Shenzhen | 1.000 | 1.000 | 1.000 | 0.628 | 1.592 | 1.000 | 1.000 | 1.000 | 0.982 | 1.023 |
| Macau | 1.136 | 1.061 | 1.000 | 1.000 | 1.000 | 1.000 | 1.000 | 1.000 | 1.000 | 1.022 |
| Hong Kong | 1.156 | 1.148 | 0.928 | 1.058 | 0.947 | 1.076 | 0.947 | 0.936 | 0.837 | 1.004 |
| Guangzhou | 1.125 | 0.689 | 1.379 | 1.285 | 0.823 | 1.112 | 0.869 | 0.999 | 0.670 | 0.995 |
| Dongguan | 1.053 | 0.890 | 0.948 | 1.393 | 1.068 | 0.911 | 1.179 | 0.874 | 0.535 | 0.983 |
| Zhuhai | 1.267 | 0.542 | 1.747 | 0.969 | 0.945 | 0.927 | 1.031 | 0.758 | 0.604 | 0.977 |
| Zhongshan | 1.279 | 1.042 | 0.816 | 0.797 | 1.014 | 1.006 | 1.159 | 0.958 | 0.677 | 0.972 |

Supplementary Table 2: The SECH under Malmquist index for the 11 cities in GBA, 2010-2019
